# Prospective Biomarkers of SARS-CoV-2 Vaccine Seroconversion in Patients with Haematological Malignancies

**DOI:** 10.3390/vaccines14030201

**Published:** 2026-02-25

**Authors:** Sophie C. Hamann, Katie E. Lineburg, Louise Ng, Annabel Waugh, Stuart Olver, Justine Leach, Christine Bristow, Jyothy Raju, Laetitia Le Texier, Pauline Crooks, Corey Smith, Kristyan Guppy-Coles, Kirk Morris, Michelle Spanevello, Siok-Keen Tey, Andrea S. Henden

**Affiliations:** 1 Queensland Institute of Medical Research Berghofer, Herston, QLD 4006, Australia; 2 Queensland Immunology Research Centre, Brisbane, QLD 4000, Australia; 3 Cancer Care Services, Royal Brisbane and Women’s Hospital, Herston, QLD 4006, Australia; 4 Faculty of Health, Medicine and Behavioural Sciences, University of Queensland, St Lucia, QLD 4067, Australia

**Keywords:** SARS-CoV-2, vaccination, haematological malignancy, immune responses

## Abstract

Background: SARS-CoV-2 vaccination is crucial for protecting against severe COVID-19 disease; however, patients with haematological malignancies (HM) respond poorly to vaccination due to immunosuppression driven by chemotherapy, targeted cell depletion, and immune dysregulation. We sought to define novel biomarkers that predict effective vaccination in patients with HM. Methods: HM patients and healthy controls received SARS-CoV-2 vaccines and were followed for six months post-vaccination. Virus-specific humoral and cellular immune responses were analysed in serum and whole blood pre- and post-vaccination, and serum proteomics was analysed pre-vaccination to identify potential biomarkers for vaccine response. Results: HM patients displayed delayed antibody seroconversion, and 37.5% failed to seroconvert. Baseline proteomic and cellular immune profiles revealed that T-cell-associated chemokines CXCL13 and CRTAM were differentially expressed, with decreased levels seen in vaccine non-responders. Vaccine response was also associated with a reduced frequency of circulating monocytes, greater numbers of B-cells, and a trend toward greater numbers of CD4+ helper cell phenotypes, including T peripheral helper cells pre-vaccination. In vitro generation of COVID-19-specific T-cells from a subset of participants trended towards increased cytotoxic CD4+ and CD8+ T-cell activity in seroconverters and dysfunctional COVID-19-specific T-cell responses in non-seroconverters. Conclusions: These results suggest that HM patients have impaired T-cell immunity, and non-responders may be identified by low levels of serum CXCL13 and CRTAM. This allows for the identification of at-risk patients who would benefit from alternative COVID-19 prophylaxis strategies.

## 1. Introduction

SARS-CoV-2 (which causes the disease COVID-19) is the respiratory virus responsible for the most recent global pandemic, causing over 7 million deaths worldwide [1]. In most healthy individuals, SARS-CoV-2 infection results in mild disease, characterised by cold and flu-like symptoms, such as a sore throat, fever, cough, and rhinitis [2]. However, immunocompromised patients are at a much higher risk of experiencing severe infection or even death, which occurs in up to 20% of patients with haematological malignancies (HM) [3]. Another problem is the persistent shedding of the virus, which leaves patients infectious for longer, increases the risk of transmission to others [4], and complicates delivery of ongoing care. Infection with SARS-CoV-2 in haematopoietic stem cell transplant (HSCT) recipients may last for approximately two weeks; however, patients can still test positive for up to five weeks from initial diagnosis [5,6]. Patients with B-cell lymphomas were also found to have persistent SARS-CoV-2 infection, particularly in those with impaired CD8+ T-cell immunity [7]. Additionally, prolonged infection has been demonstrated to lead to viral mutations and the emergence of variants [8].

Recent studies have reported the importance of B-cells and long-lived bone marrow plasma cells for protecting against severe disease in an immunocompetent setting [9,10,11]. Supporting this, diminished B-cell responses and virus-specific antibody production have been associated with increased hospitalisation and mortality in SARS-CoV-2-infected patients with blood cancers and disorders, in both adult and paediatric populations [7,12,13]. T follicular helper (Tfh) cells and plasmablasts were also reduced in patients with HM [12]. Interestingly, elevated CD8+ T-cell numbers were associated with improved survival, even in patients receiving anti-CD20 therapy [12], suggesting that CD8+ T-cells may be crucial for protecting against severe disease in these immunocompromised patients.

Current treatments for SARS-CoV-2 infections include budesonide, remdesivir, and nirmatrelvir/ritonavir (Paxlovid), with tocilizumab, baricitinib, and dexamethasone indicated for severe disease [14,15,16,17]. However, the efficacy of these varies, with viral variants responding differently to each treatment modality. A promising treatment for immunocompromised patients is virus-specific T-cell (VST) therapy. In immunosuppressed patients with HM, VST therapy reduced viral load and boosted SARS-CoV-2-specific T-cell responses [18]. Additionally, studies have shown that post-HSCT patients with persistent SARS-CoV-2 viral infection and who are resistant to standard anti-viral therapy had viral clearance within two months after treatment with allogeneic and autologous VSTs. Patients remained PCR negative for the virus at six months post-treatment and had induction of antibody- and virus-specific CD4+ memory T-cell responses [19,20].

An important strategy for preventing severe disease is vaccination. SARS-CoV-2 vaccination is recommended for HM and HSCT recipients [17,21,22,23]. However, recent studies demonstrate that the response to vaccination is sub-optimal. Among a population of immunosuppressed individuals, patients with HM showed the highest rate of vaccine failure after up to three doses [24]. Patients with lymphoid malignancies showed lower antibody responses to vaccination compared to those with myeloid cancers [25]. Not surprisingly, patients who received B-cell-depleting therapies had impaired antibody responses following vaccination, especially those on active treatments [24,25]. Despite no changes in COVID-19-related mortality observed in vaccinated compared to unvaccinated HM patients, vaccination significantly reduced the risk of hospitalisation in HM patients with break-through infections [26]. Vaccinated HSCT patients showed decreased seroconversion and T-cell responses compared to healthy controls, where only 86% of patients developed adequate antibody levels [27,28,29,30]. Impaired antibody responses to vaccination were associated with lymphopenia, active graft-versus-host disease, low IgG levels, and immunisation within the first year of transplant [30,31]. These low response rates to SARS-CoV-2 vaccination demonstrate limited immunogenicity in HM and HSCT patients who may require additional boosters and/or alternative treatments [28].

Here we have performed an exploratory study analysing cellular and humoral immune responses to SARS-CoV-2 vaccination in a cohort of immunosuppressed patients. We demonstrate that patients with HM have a reduced ability to seroconvert compared to healthy controls and show chemokine dysregulation prior to vaccination. Immunosuppressed patients who do not respond to vaccination have reduced cytotoxic virus-specific T-cell expansion, and proteomic analysis showed a defect in T- and B-cell immunity in these patients compared to HM patients that did seroconvert, which may be used as a predictive marker to indicate vaccine failure.

## 2. Materials and Methods

### 2.1. Patient Recruitment and Vaccination

**Figure 1:**
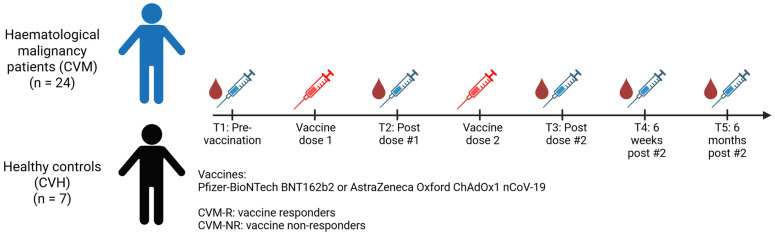
Timeline of sample collection and vaccination.

The study was performed in accordance with the Declaration of Helsinki, the International Conference on Harmonisation Good Clinical Practice Guidelines, the NHMRC National Statement on Ethical Conduct in Human Research 2007, and the Australian Code for the Responsible Conduct of Research, 2018. Ethics were approved by QIMR Berghofer and the Royal Brisbane and Women’s Hospital (Ethics number: HREC/2021/QRBW/73535). The COVID-19 vaccine administered to patients was either the Pfizer-BioNTech BNT162b2 or the AstraZeneca Oxford ChAdOx1 nCoV-19 (AZD1222) vaccine pursuant to the Australian Government Department of Health, Disability and Ageing provision. Inclusion details for the study included participants aged 18 years and over who were able to provide voluntary informed consent and who planned to receive a COVID-19 vaccine of any variety. Cohort A included patients who had any haematological malignancy and had received treatment with a B-cell-targeted therapy or had received a HSCT. Cohort B included patients who did not have a diagnosis of haematological malignancy or any other cancer, and who had not received any cancer or immunosuppressing therapy. The clinical study recruited participants between February 2021 and December 2022. The timeline of sample collection and vaccination is outlined in Figure 1. Serum and peripheral blood mononuclear cell (PBMC) preparations were extracted from blood collected in serum separator and Lithium Heparin tubes respectively.

### 2.2. IgG Measurements

IgG antibody levels from serum were determined using the AdviseDx SARS-CoV-2 IgG II assay (Abbott Laboratories, Chicago, IL, USA) following the manufacturer’s instructions. This is a chemiluminescent microparticle immunoassay that detects IgG antibodies directed to the receptor-binding domain of the S1 subunit of the spike (S) protein and produces a qualitative and semi-quantitative result. IgG levels below 50 AU/mL were considered negative as per the manufacturer’s protocol.

### 2.3. COVID-19-Specific T-Cell Expansion

SARS-CoV-2-specific T-cells were expanded using a published method [32]. Briefly, peripheral blood mononuclear cells (PBMCs) from the post-vaccine dose 1 timepoint were incubated with overlapping peptide pools directed to the S protein and grown in RPMI-1640 media containing 1% penicillin–streptomycin (Gibco, Waltham, MA, USA) and 10% foetal bovine serum, supplemented with recombinant IL-2 every 2-3 days thereafter. T-cells were harvested on day 14 and assessed for antigen specificity using an intracellular cytokine assay.

### 2.4. Cytokine Analysis

Cytokines were measured in serum collected from participants at defined timepoints post-vaccination (Figure 1). Additionally, an immediate whole blood assay was used to determine SARS-CoV-2-antigen-specific T-cell responses using a published method [32,33]. Briefly, whole blood was incubated with two overlapping peptide pools representing the S protein for up to 24 h. Positive (phytohaemagglutinin mitogen [PHA]) and negative (no peptide) controls were included. Following incubation, plasma was harvested for cytokine analysis. A cytometric bead array (CBA) was used to quantify cytokines in both the serum samples and the supernatant derived from the whole blood using the BD Flex-sets. Samples were acquired on a BD LSR Fortessa using FACSDiva software (v3.0, BD Bioscience, San Jose, CA, USA) and analysed using FCAP array software (v3.0, BD Bioscience, San Jose, CA, USA). The following cytokines were quantified: IL-1β, IL-2, IL-4, IL-5, IL-6, IL-8, IL-9, IL-10, IL-13, IL-17A, TNF, and IFNγ. For whole blood analysis, cytokine levels in the negative control were subtracted from the corresponding test conditions to account for nonspecific, spontaneous cytokine production.

### 2.5. Flow Cytometry

Spectral flow cytometry analysis: PBMCs from the pre-vaccination timepoint were isolated and stained with a high-dimensional flow cytometry panel for the phenotypic assessment of immune populations and T-cell subsets (Table S1). Frozen PBMCs were rapidly thawed at 37 °C with RPMI media. Cells were then washed with PBS and stained with a live/dead blue viability dye (Invitrogen, Waltham, MA, USA) for 15 min using a published method [32]. Following this, cells were incubated with true stain monocyte blocker (BioLegend, San Diego, CA, USA), Brilliant Stain Buffer Plus (BD Horizon, Milpitas, CA, USA), and anti-CCR7 antibody for 10 min to reduce competitive binding. Cells were then stained with anti-CCR6, CCR5, CXCR5, and CXCR3 for 5 min, followed by anti-TCRγδ for 10 min and the remaining antibodies for a further 30 min. Antibodies were then washed off, and cells were fixed with 1% paraformaldehyde for 20 min. All incubations were performed at room temperature in the dark. Samples were acquired on a Cytek Aurora spectral flow cytometer on the same day. Data was analysed using FlowJo software (v10.9). Gating for the T-cell subset analysis was performed as outlined in Supplementary Figure S3.

Cytotoxic flow assays: For intracellular staining, expanded T-cells harvested at D+14-17 of in vitro culture were re-called with SARS-CoV-2 antigen peptide pools and incubated for 4 h with GolgiPlug, GolgiStop, and anti-CD107a-FITC (BD Bioscience, San Jose, CA, USA), as previously reported [32]. Following stimulation, cells underwent surface marker antibody staining for 30 min before being fixed and permeabilised using the BD Cytofix/Cytoperm kit (BD Bioscience, San Jose, CA, USA) as per the manufacturer’s instructions. Following fixation, cells were stained with intracellular markers as previously described [32], and they were analysed on a BD LSR Fortessa with FACSDiva software (v8) and analysed using FlowJo software (v10.9).

### 2.6. Serum Proteomic Analysis

The protein profile was analysed in serum samples from the pre-vaccination timepoint to determine a potential biomarker for vaccine response using the Olink multiplex proximity extension assay (PEA). The immuno-oncology panel was chosen as it includes the measurement of 92 proteins involved in various aspects of immune responses. Samples were distributed in a 96-well plate and shipped frozen to the Australian Genome Research Facility for analysis. The serum samples were incubated with antibodies carrying unique DNA tags that bind to their respective protein within the sample. The DNA tags, when in close proximity, hybridise and form DNA templates through extension by DNA polymerase. These are then amplified and quantified using quantitative polymerase chain reaction. Data is then processed through software reporting the relative concentrations of the proteins on a log2 scale, called normalised protein expression (NPX).

### 2.7. Statistical Analysis

Data was analysed using GraphPad Prism software (v10.1.2). Data was tested for normality using the D’Agostino & Pearson normality test. Depending on data distribution, either a Welch’s *t* test, multiple unpaired *t* test, or Mann–Whitney U test was performed. A *p* value < 0.05 was considered statistically significant.

## 3. Results

### 3.1. Study Population

In total, 31 participants were included in this exploratory study, 24 of whom were treated for haematological malignancies and 7 of whom were healthy controls (Table 1). In patients with haematological malignancies (referred to as CVM), 58.2% were male, and the average age was 56.7 years. Participants had a range of HM, including non-Hodgkin lymphoma (NHL; 33.3%), myeloma (25%), acute myeloid leukaemia (AML; 16.7%), acute lymphoblastic leukaemia (ALL; 8%), HL (Hodgkin lymphoma; 4%), chronic myeloid leukaemia (CML; 4%), and others (juvenile myelomonocytic leukaemia [JMML], essential thrombocythemia [ET], and systemic sclerosis [SS]; 8%). Treatments included allogeneic HSCT (alloHSCT; 33.3%), autologous stem cell transplantation (ASCT; 12.5%), B-cell-depleting therapy with or without chemotherapy (33.3%), plasma cell targeted therapy (8.3%), and immunomodulatory drugs (IMiDs; 12.5%). Healthy control participants (referred to as CVH) were 71.4% female with an average age of 34 years. Participants received either the Astra-Zeneca ChAdOx1 nCoV-19 “Vaxzevria” (33.3% and 14.3% CVM and CVH respectively) or the Pfizer BioNTech BNT162b2 “Comirnaty” (66.6% and 85.7% CVM and CVH respectively) vaccine. Two doses of the vaccine were administered at approximately one month apart, with samples taken at five timepoints (pre-vaccination, post-vaccine dose 1, post-vaccine dose 2, 6 weeks after last dose, and 6 months after final dose). All patients were SARS-CoV-2 naïve prior to vaccination.

### 3.2. Immunocompromised Patients Show Delayed Antibody Production

**Figure 2:**
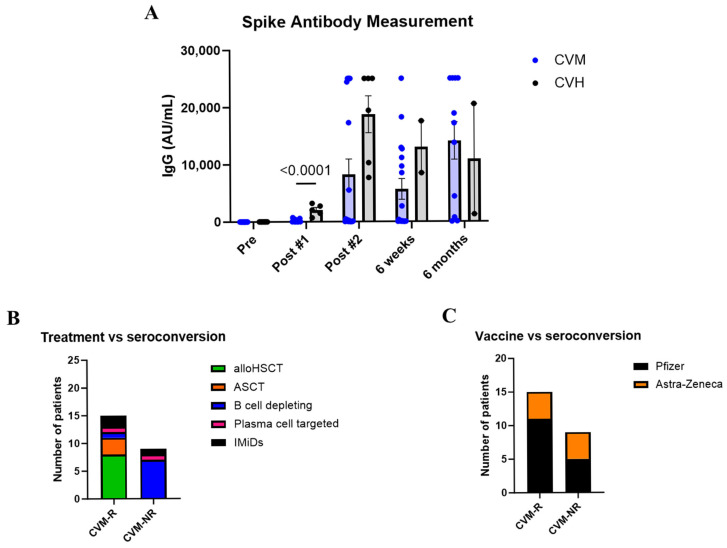
Antibody responses to vaccination. (**A**) IgG responses to the spike protein of SARS-CoV-2 in patients with haematological malignancy and healthy controls over time after vaccination. (**B**) Effect of treatment type on seroconversion status in immunosuppressed patients. (**C**) Effect of vaccine type on seroconversion status in immunocompromised patients. Statistical significance was determined by a multiple unpaired *t* test. Data presented as mean ± SEM. CVM-R = HM patient vaccine responders. CVM-NR = malignant patient vaccine non-responders.

To assess seroconversion after vaccination, IgG responses to the spike protein of SARS-CoV-2 were measured in serum. As expected, both groups had no detectable IgG prior to vaccination. Following the first dose, CVM patients had significantly decreased IgG levels compared to CVH participants (*p* = <0.0001). A trend of reduced spike-specific IgG was also seen after the second vaccination, as well as 6 weeks post second dose, where CVM patients still had lower IgG levels compared to healthy controls (*p* = 0.1831 and *p* = 0.5092 respectively) (Figure 2A). This demonstrates that immunocompromised patients have a delayed antibody response to vaccination. However, by 6 months post-vaccination, immunocompromised patients and healthy controls who responded to vaccination had similar levels of IgG (*p* = 0.8253, Figure 2A). This may be due to the fact that patients who received time-limited therapies (such as autologous and allogeneic transplants, as well as those treated for lymphomas) had increased time from administration of the last immunosuppressive treatment, permitting a greater degree of immune reconstitution. Importantly, only 62.5% of immunocompromised patients seroconverted following vaccination (defined as detectable IgG by the final timepoint), versus 100% of healthy controls. From here on in, CVM patients were split into responders (CVM-R) and non-responders (CVM-NR) for further analysis. Additionally, as IgG levels were comparable between CVM and CVH cohorts at 6 months post-vaccination, further analysis was only performed on the discriminating timepoints up to 6 weeks post-vaccination. Among immunosuppressed patients, those receiving B-cell-depleting therapies, as opposed to alloHSCT or ASCT, were associated with reduced seroconversion (Figure 2B), showing treatment-specific effects on seroconversion. However, due to the small cohort size, it is difficult to decipher whether seroconversion was directly correlated with treatment modality. Finally, vaccine type did not affect the ability of CVM patients to seroconvert (Figure 2C).

### 3.3. No Differences Were Observed in Cytokine Levels Between CVM-R and CVM-NR

**Figure 3:**
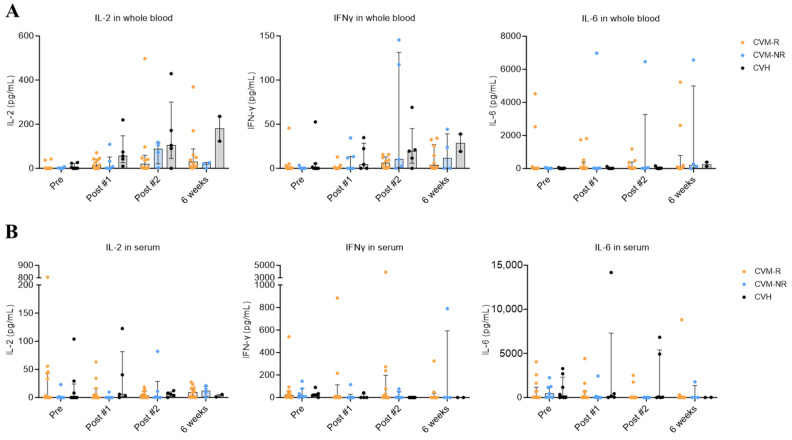
Whole blood and serum cytokine responses after SARS-CoV-2 vaccination. (**A**) IL-2, IFNγ, and IL-6 responses in SARS-CoV-2 peptide-stimulated whole blood from CVM and CVH patients. (**B**) IL-2, IFNγ, and IL-6 responses in serum of CVM and CVH patients. Statistical significance was determined by a multiple unpaired *t* test. No statistically significant differences were observed. Data presented as median with interquartile range (IQR).

Cytokine responses in all participants were analysed using an established whole blood assay [32,33] to identify virus-specific T-cell responses, as well as in serum reflecting global cytokine responses. Statistically significant differences in T-cell-specific and global cytokine responses were not observed in this study. Cytokine detection levels were not significantly different between CVH and CVM cohorts, regardless of their seroconversion status. The lack of any statistically significant differences is likely driven by the small sample size and the impact of individual outliers (Figure 3A,B).

No striking differences were noted in the remaining cytokines for either the whole blood assay (Figure S1) or in the serum (Figure S2).

### 3.4. CXCL13 and CRTAM as Prospective Biomarkers of Vaccine Response in HM Patients

**Figure 4:**
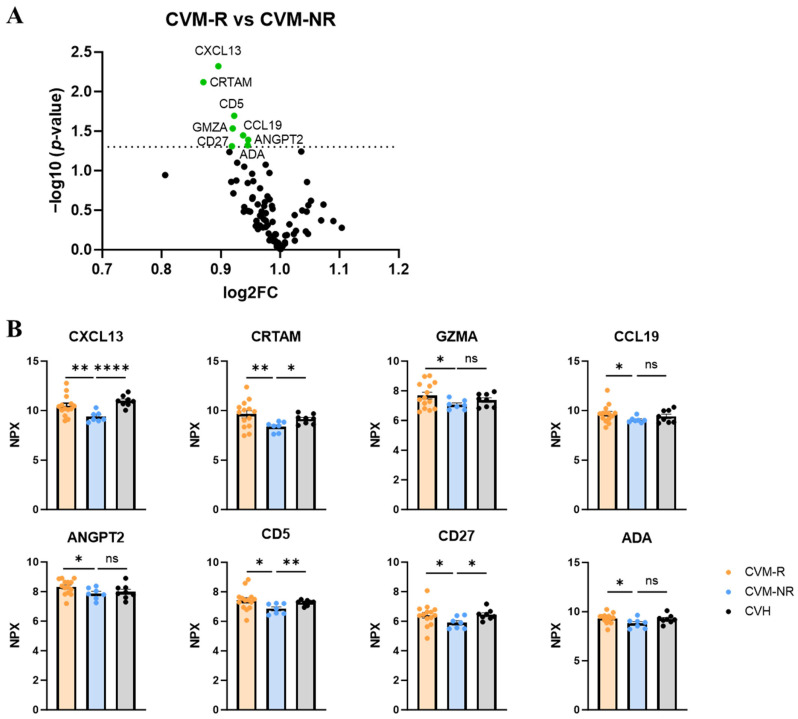
Proteomic analysis in pre-vaccination serum of responders and non-responders. (**A**) Volcano plot of protein changes in vaccine non-responders compared to responders. Dotted line indicates statistical significance. (**B**) Normalised protein expression (NPX) of proteins in vaccine responders, non-responders, and healthy controls. Statistical significance was determined by either a Mann–Whitney U Test or a Welch’s *t* test, depending on data normality. Data presented as mean ± SEM. * *p* < 0.05, ** *p* < 0.01, and **** *p* < 0.0001. ns: not significant.

To identify prospective biomarkers of seroconversion, we performed proteomic analysis using the Olink immuno-oncology panel on serum from all participants prior to vaccination. Within CVM patients, a number of proteins were significantly differentially expressed in non-responders (CVM-NR) compared to responders (CVM-R). These included CXCL13 (Chemokine (C-X-C motif) ligand 13), CRTAM (Class-I MHC-restricted T-cell-associated molecule), CD5, GZMA (granzyme A), CCL19 (C-C motif chemokine ligand 19), CD27, ANGPT2 (angiopoietin-2), and ADA (adenosine deaminase) (Figure 4A,B). CXCL13 was the most significantly upregulated protein in CVM-R compared to CVM-NR (*p* = 0.0097). This protein is a B-cell chemotactic, and thus a decrease in this protein in CVM-NR suggests an impact on humoral immune responses to vaccination. CRTAM also stood out as significantly decreased in CVM-NR compared to CVM-R patients (*p* = 0.0076). This protein is involved in T-cell cytotoxicity, and this finding is relevant to our subsequent analysis of dysfunctional T-cell immunity in CVM-NR participants. The expression of these two proteins was also significantly reduced in CVM-NR compared to CVHs, whereas there was no difference in CVM-R compared to CVH (Figure 4B). This validates the specificity and clinical relevance of these proteins as biomarkers for vaccine response in patients with malignancy. Additionally, CCL19 is involved in T-cell trafficking, while CD5, CD27, GZMA, and ADA are involved in T-cell activation, proliferation, cytotoxicity, and survival. In summary, these proteomic findings demonstrate reduced protein expression in CVM-NR patients in adaptive immune response pathways, which are associated with subsequent vaccine failure.

### 3.5. Immunophenotyping of Immune Cell Subsets in HM Patients Prior to Vaccination

**Figure 5:**
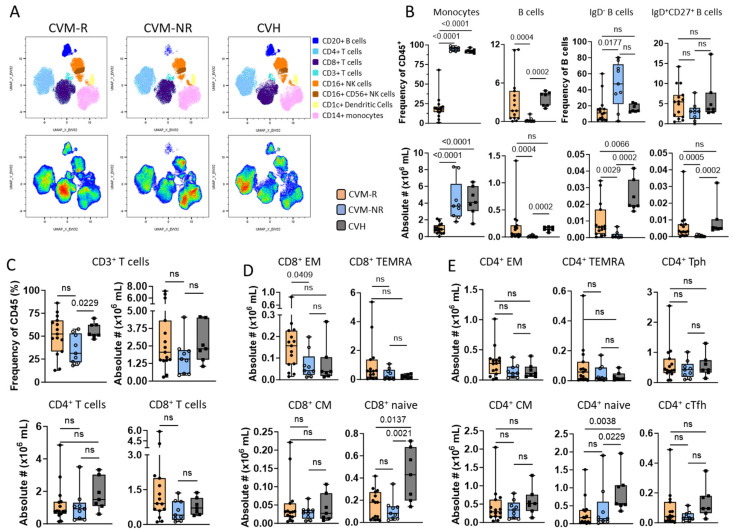
Immunophenotyping of immune cell subsets in HM PBMCs pre-vaccination. (**A**) Uniform Manifold Approximation and Projection (UMAP) clustering overlays of HM patients comparing CVM-R (left, n = 15) and CVM-NR (right, n = 9). Populations were identified by surface expression of defined markers. (**B**–**E**) Box and whisker plots comparing frequency (%) and absolute number (#×10^6^/mL) of CD45+ cells between CVM-R, CVM-NR, and CVH. (**B**) Non-T-cell immune populations, including CD45+ CD14+ monocytes, CD19+ CD20+ B-cells, and B-cell subsets: IgD- and IgD+ CD27+. (**C**) Frequency (%) and distribution of CD4+ and CD8+ T-cells (CD3+ CD56-). (**D**) CD8+ memory T-cell subsets defined by CD45RA and CCR7; effector memory (CCR7- CD45RA-) naïve (CCR7+ CD45RA+), central memory (CCR7+ CD45A-), and terminal effector (CCR7- CD45RA+). (**E**) CD4+ memory T-cell subsets, as for CD8+ and including helper T-cell subsets, conventional T follicular helper (CXCR5+ PD-1+), and T peripheral helper (CXCR5- PD-1+). Statistical significance was determined by Mann–Whitney U Test. ns: not significant.

To interrogate the composition of HM patient immune populations prior to vaccination, PBMCs were antibody-stained using a 35 colour immune profiling panel (Table S1). This flow cytometry approach was used to compare patient PBMCs at the pre-vaccination timepoint, comparing CVM-R with CVM-NR and CVH patient subsets to identify compositional differences in their baseline immune cell composition (Figure 5A and Figure S3).

High-dimensional analysis identified a significantly greater monocyte frequency and number (*p* < 0.001) in CVM-NR and CVH patients compared to CVM-R (Figure 5B). In the lymphocyte compartment, CVM-NR demonstrated a significantly lower frequency and number of B-cells compared to CVM-R (*p* < 0.004) and CVH (*p* = 0.0002) (Figure 5B). Within this reduced B-cell compartment, CVM-NR displayed a significant reduction in both IgD- B-cell frequency (*p* = 0.014) and number (*p* = 0.0029) compared to CVM-R and CVH. While IgD+CD27+ B-cell frequency was not significantly different, the absolute numbers were reduced in CVM-NR compared to CVM-R (*p* = 0.0005) and CVH (*p* = 0.0002) (Figure 5B).

In the T-cell compartment, absolute numbers of total CD3+ (CD56-) T-cells trended lower in CVM-NR, including both CD4+ T and CD8+ T-cell subsets (Figure 5C). The number of CD8+ EM T-cells was significantly reduced in CVM-NR, with CD8+ Naïve, CD8+ CM, and CD8+ TEMRA (CCR7-CD45RA+) T-cell subsets all trending lower in CVM-NR compared to CVM-R (Figure 5D). Similar reductions were noted in multiple CD4+ T-cell subsets, including T follicular helper (Tfh: PD-1+CXCR5+), T peripheral helper (Tph: PD-1+CXCR5-), central memory (CM: CCR7+CD45RA-), and effector memory (EM: CCR7-CD45-) populations (Figure 5E). Both CD8+ and CD4+ Naïve T-cell numbers were significantly higher in healthy controls.

The frequencies of CD4+ regulatory T-cells (Treg) were significantly decreased in CVM-NR patients in comparison to CVH (*p* = 0.0052), while CVM-R were significantly reduced in both frequency (*p* = 0.0016) and absolute number (*p* = 0.0179) in comparison to CVH. However, there were no differences observed in the frequency or number of NK populations, including early NK (CD56^bright^ CD16-), mature NK (CD56^dim^CD16+), or terminal NK (CD56-CD16+); and there were no differences in frequency or numbers of CD3+CD56+ (NKT) or TCRγδ+ T-cells when we compared CVM-NR and CVM-R HM patient subsets (Figure S4).

### 3.6. SARS-CoV-2-Specific T-Cells from Vaccine Responders Show Trends Towards Increased Cytotoxic Activity

**Figure 6:**
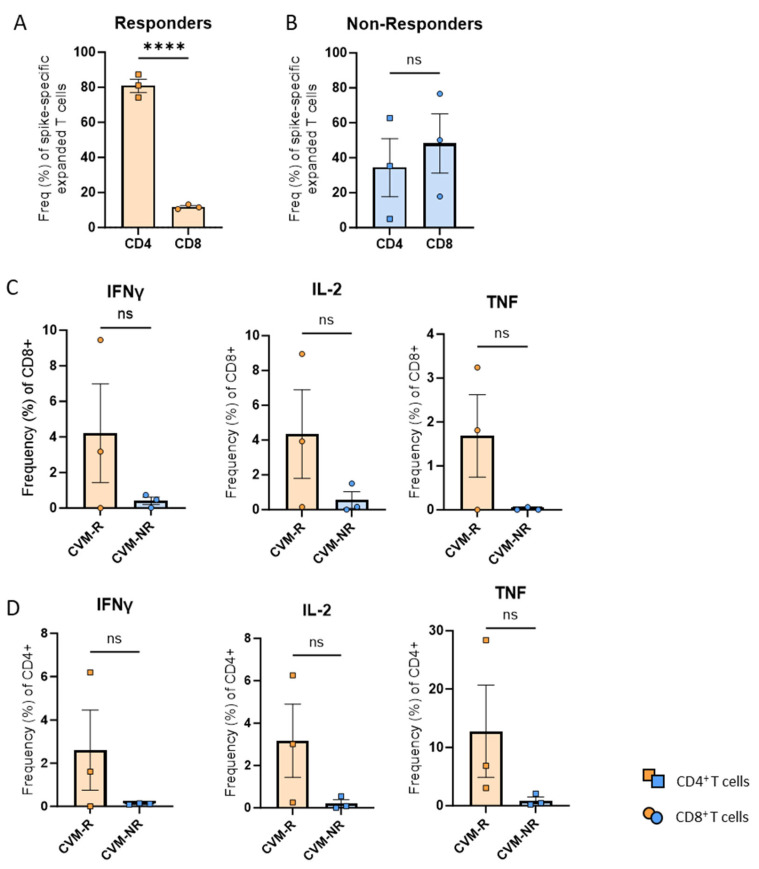
COVID-19-specific T-cell responses in vaccine responders and non-responders. (**A**) Frequency of spike-specific CD4+ and CD8+ T-cells in vaccine responders. (**B**) Frequency of spike-specific CD4+ and CD8+ T-cells in vaccine non-responders. (**C**) Frequency of cytokine-producing CD8+ T-cells. (**D**) Frequency of cytokine-producing CD4+ T-cells. Data presented as mean ± SEM. Statistical significance was determined using an unpaired *t*-test. **** *p* < 0.0001. ns: not significant.

To explore the functionality of T-cells derived from CVM-R versus CVM-NR, PBMCs collected at the second timepoint, after the 1st dose of vaccine, from six patients were identified. Three responders were selected as those who seroconverted and had numerically high cytokine responses in the COVID-19–peptide-stimulated whole blood assay (Figure 3A) versus three non-responders who had not seroconverted and had numerically poorer responses in the whole blood assay. PBMCs were stimulated with SARS-CoV-2 peptide pools (directed to the spike protein) and expanded in culture as previously described [32]. PBMCs were then stained with IL-2, TNF, and IFNγ to determine functionality. Interestingly, responders had a significantly greater expansion of CD4+ T-cells compared to CD8+ T-cells (*p* < 0.0001) (Figure 6A), and compared to non-responders, which showed a more similar expansion of both CD4+ and CD8+ T-cells (*p* = 0.5916) (Figure 6B). This may be due to more immunodominant CD4+ T-cell epitopes against the spike protein [34]. CD8+ T-cells in responders trended towards elevated IFNγ, IL-2, and TNF expression compared to non-responders (Figure 6C). A similar trend was also observed in the CD4+ T-cell compartment (Figure 6D). Importantly, due to variability between patients combined with low sample size, statistical significance was not reached, and the observations made are trends only. A larger cohort may shift these trends into significance. These findings suggest that cytotoxic T-cell activity from both CD4+ and CD8+ T-cells is potentially impaired in immunosuppressed patients who do not subsequently seroconvert, highlighting the importance of T-cell immunity in vaccine efficacy.

## 4. Discussion

Vaccination is an effective strategy to reduce illness and death due to COVID-19; however, patients with HM are likely to receive therapies that reduce or prevent effective vaccination. In the era of COVID-19 endemicity, we require biomarkers to identify patients who will not be protected by vaccination, in order to offer alternative strategies to reduce disease severity and death. Studies in both healthy and patient cohorts demonstrate that not all individuals respond equally to vaccination, and this varied immunogenicity has been linked to multiple co-factors that impact both antibody and T-cell responsiveness [35,36,37,38,39]. The impact of these variables in HM patients is conferred by the mechanisms of standard-of-care interventions, including B-cell-depleting therapies, chemotherapies, and more [40,41]. These complex immune insults highlight how heterogeneous the HM patient population is and highlight the urgent need for biomarkers that are indicative of a patient’s individual risk when it comes to vaccine responsiveness.

This study examined a small cohort of 24 patients with a haematological malignancy compared to healthy control participants and evaluated both antibody and T-cell responses to assess the priming of SARS-CoV-2-specific immunity to the COVID-19 vaccine. A comprehensive baseline immunological survey was performed to identify biomarkers for HM individuals at risk for failing to generate immunogenicity in response to COVID-19 vaccination. This study was carried out in Australia prior to any widespread transmission of SARS-CoV-2.

COVID-19 vaccination resulted in reduced responsiveness in HM participants compared to healthy controls (Figure 2A). Both cohorts displayed increasing S-specific IgG titres over the course of COVID-19 vaccination; however, responses were significantly reduced in HM patients compared to healthy controls after the first dose of the COVID-19 vaccine. S-specific IgG titres in CVH peaked after the second vaccine dose, displaying a significant (*p* < 0.0001) increase in antibodies compared to after the first dose. In comparison, CVM patients demonstrated a more delayed increase in S-specific IgG titres, with the highest titres identified at the 6 month timepoint. CVM titres reached a maximal detection level at 6 months post commencement of vaccination, but failed to match the peak post-vaccine dose 2 response titres observed in CVH participants. Previously, large cohort studies have demonstrated that cancer patients display varied COVID-19 vaccine responses, highlighting that patients with HM demonstrate reduced responsiveness in comparison to those with solid cancers [42,43,44]. Patients with HM were shown to benefit from additional COVID-19 vaccine doses at shorter intervals in order to achieve comparable SARS-CoV-2-specific immunity to solid cancer patients [45,46].

We further identified 62.5% (15/24) as responders (CVM-R), defined by the detection of SARS-CoV-2-specific IgG antibodies, and 37.5% (9/24) as non-responders (CVM-NR), defined by failure to mount a detectable SARS-CoV-2-specific antibody response over the 6-month post-vaccine study monitoring period (Figure 2A). Participants recruited early in the study received an AstraZeneca vaccine, and those recruited later received Pfizer; however, seroconversion was seen equally following both vaccines (Figure 2C), which is consistent with reports from similar studies [42]. However, we observed a treatment-specific effect on seroconversion within CVM patients, where the majority of patients receiving B-cell depletion were unable to mount SARS-CoV-2-specific IgG antibodies by 6 months post-vaccination (Figure 2B), which is consistent with previous reports [47,48]. The rate of seroconversion in patients who had received an alloHSCT is reassuring, as both post-transplant immunosuppression and graft-versus-host disease impair cellular and humoral immune responses. Failure to seroconvert was also seen in recipients of immunomodulatory drugs and plasma cell-targeting monoclonal antibodies. These treatments are used in a continuous fashion in plasma cell myeloma, suggesting patients with this malignancy may have an increased risk of vaccine failure. Another Australian study (SerOZNet study [42]) examined COVID-19 vaccine responses in a larger cohort study comparing HM with SOT patient cohorts and, similarly, identified the minimal impact of other co-factors, including age, steroids, and cytotoxic chemotherapy agents, on vaccine responsiveness.

The importance of T-cell responses in controlling COVID-19 and preventing severe disease is well established, though, and we therefore sought to interrogate the presence of SARS-CoV-2-specific T-cell responses using a published whole blood assay (WBA) (Figure 2) [32,33]. Consistent with WBA monitoring performed in healthy vaccinated responders [49] and cancer patients [42], we observed a trend towards increased expression of IL-2 and IFNγ within vaccine responders compared to the non-responding subset. Levels of other cytokines, including IL-6, however, were difficult to interpret in our small dataset due to the influence of outlying values, often 1 log or greater in quantitation.

Proteomic analysis using the Olink immuno-oncology panel identified significantly decreased expression of several proteins in CVM non-responders compared to responders, including CXCL13, CRTAM, CD5, GZMA, CCL19, ANGPT2, CD27, and ADA (Figure 4). CXCL13 was identified as the most significantly decreased protein in CVM-NR compared to CVM-R. It is a chemotactic for B-cells, which is expressed on follicular dendritic cells and germinal centre Tfh cells, and can be produced by Tph cells to boost B-cell activity [50,51]. CXCL13 has been described as a plasma biomarker for germinal centre activity following infection and vaccination in both influenza and HIV [51,52,53]. Indeed, Wan et al. have shown that the magnitude and breadth of antibody- and antigen-specific T-cell responses against SARS-CoV-2 and influenza viral proteins were improved by delivering CXCL13 to lymph nodes through lipid nanoparticles, where it altered the transcriptomic profile of lymph nodes to promote the formation of germinal centres [54]. In our study, reduced serum CXCL13 in vaccine non-responders may indicate a perturbed germinal centre as well as B-cell and Tfh cell function.

CRTAM expression was also reduced in CVM-NR patients. CRTAM has an important role in antigen-specific T-cell activation, adhesion, and draining lymph node retention, primarily in CD8+ T-cells; however, a small fraction of CD4+ T-cells also express this protein [55,56,57]. This protein has been shown to bind to CADM1 (Necl-2) on dendritic cells, which upregulates its expression on T-cells and initiates CRTAM-mediated signalling to activate cytotoxic-related genes [56,58,59]. CRTAM has also been demonstrated to induce cell polarity during activation and regulate effector function in a small subset of CD4+ T-cells [55]. CD4+ T-cells that express CRTAM have been shown to secrete IFNγ and express cytotoxic T lymphocyte (CTL)-related genes, such as, for example, Eomes, granzyme B, and perforin [56]. In mice deficient in CRTAM, T-cells displayed reduced IFNγ and IL-22 production as well as impaired anti-viral immunity [57,60]. Thus, it could be hypothesised that a downregulation of this protein in vaccine non-responders in our study suggests impaired T-cell activation, either due to T-cell dysfunction or impaired antigen presentation.

Similarly, ADA and CD27 are important in activating and expanding the T-cell immune compartment. ADA has been shown to trigger CD4+ T-cell activation and proliferation, enhancing their memory and effector cell functions, as well as controlling Tfh cells through IL-6/IL-22 to provide B-cell help [61,62]. Early studies have demonstrated that CD27 was crucial for the generation of influenza-specific T-cells in the lung and lung-draining lymph nodes, while more recently, a role for this protein in protecting against Epstein–Barr virus (EBV) has been reported [63,64]. In our study, these proteins may play a role in developing a robust T-cell immune response to SARS-CoV-2 vaccination, and their reduction may lead to vaccine failure, as observed in CVM-NR patients.

Additionally, our data suggests that CVM-NR patients may have defects in their antigen presentation. CD5 has been demonstrated to be expressed on type 2 dendritic cells, where it functions to prime CD4+ helper and cytotoxic T-cells in tumours [65]. Moreover, granzyme A has been shown to induce the maturation of plasmacytoid DCs (pDCs), enhancing their functionality, leading to increased type I interferon production, and eliciting antigen-specific CD8+ T-cell responses [66]. Reductions in these proteins may indicate that vaccine non-responders fail to prime their adaptive immune system and mount virus-specific T-cell responses.

Finally, this study has identified a potential role for CCL19 in vaccine response. CCL19 is a chemotactic that recruits DCs and lymphocytes to tissues and lymphoid organs and has been used to enhance antigen-specific CD4+ and CD8+ T-cell responses [67]. This may link with poor priming and expansion of T-cells, where migration of antigen-presenting cells is impaired, and therefore, they are unable to interact with T-cells, and thus impair response to vaccination.

Most of the vaccine non-responders in our cohort were receiving B-cell-depleting therapies with attendant humoral immune cell compartment dysfunction. A reduction in these proteins in CVM-NR suggests defects in generating T-cell immunity in response to vaccination and a lack of effective T-cell-driven SARS-CoV-2 control. These proteins may serve as prospective biomarkers for effective vaccine responses. Pre-emptive identification of those unlikely to benefit from vaccination can inform alternative treatment algorithms for high-risk patients, including early intensification of therapy and consideration of adoptive cellular therapies for subsequent confirmed disease [18].

High-dimensional flow cytometry was used to compare PBMC samples at baseline in patient responders versus non-responders to reveal cellular associations with vaccine responsiveness. Importantly, our cohort comprised 41.6% of patients who had been treated with a B-cell-depleting (33.3%) or plasma cell-targeting (8.3%) therapy, and eight of nine non-responders had received these therapies. The impact of these therapies was evident in spectral analysis, demonstrating a significant difference in total B-cell frequency and number between CVM-R and CVM-NR patient subsets (Figure 4). B-cell subset analysis, however, revealed a significant increase in the frequency of IgD-B-cells (*p* = 0.0177) within the CVM-NR patient subset compared to CVM-R.

Within the pre-vaccine T-cell compartment, non-responders demonstrated reduced total CD3+ T-cells encompassing lower cell numbers in both CD4+ and CD8+ T-cell subsets compared to responders. Within this contracted T-cell compartment, CVM-NR displayed a significantly increased frequency of CD4+ T-cells compared to CVM-R, which demonstrated equal proportions of CD4+ to CD8+ T-cell frequencies. While no significant differences were observed in the frequencies of CD4+ Treg, Tfh, or Tph populations, absolute numbers revealed a trend toward reduced Tfh and Tph numbers in CVM-NR compared to CVM-R. This trend mirrors the significant reduction in associated chemokine CXCL13 [54,68], which was also reduced in CVM-NR. In the memory compartment, absolute numbers of CD8+ EM T-cells were significantly reduced (*p* = 0.0409), and CD4+ EM T-cells similarly trended lower in CVM-NR compared to CVM-R. Whilst not reaching significance in our small study, the trend of a reduction in Tph cells represents a biologically plausible cellular biomarker for response, linked with our observations regarding CXCL13.

The capacity to prime SARS-CoV-2-specific T-cell memory in patients who have received treatment for haematological malignancy was further assessed through the generation and assessment of cytotoxic effector function in expanded S-specific T-cells derived from CVM-R (n = 3) and CVM-NR (n = 3) patients after one dose of vaccine. Expanded S-specific T-cells from CVM-R were predominantly CD4+ T-cells (median ~80%), while CVM-NR expansions displayed similar proportions of CD4+ and CD8+ T-cells. Considering that multiple studies now evidence a majority of S-specific T-cell epitopes prime CD4+-specific responses in primary infection and vaccination [34,49,69,70,71], these findings suggest that CVM-Rs primed the expansion of functional (IFNγ, IL-2, and TNF-producing) S-specific CD4+ memory T-cell responses more effectively than CVM-NR patients, whose expanded T-cells failed to generate comparable cytotoxic cytokine responses. However, validation analyses in larger cohorts are required to confirm our findings.

## 5. Conclusions

In summary, we have shown that patients with haematological malignancies display reduced humoral responses and impaired T-cell immunity after SARS-CoV-2 vaccination. Receipt of B-cell-depleting therapies was most commonly associated with failure to seroconvert. Vaccine non-responders displayed reduced T-cell activation, proliferation, and cytotoxicity compared to responders, and we identified CXCL13 and CRTAM as prospective biomarkers for failed seroconversion, along with a non-significant trend in the biologically relevant Tph T-cell subset. Whilst requiring further validation in larger cohorts, these findings present a practical way to identify high-risk patients who remain at risk despite the declaration of the end of the COVID-19 global health emergency in May 2023 [72].

## Figures and Tables

**Table 1:** Patient characteristics.

	Treated Malignancy (CVM)			Healthy Controls (CVH)			
Number	24			7			
Gender	Male	14	58.3%	Male		2	28.6%
	Female	10	41.7%	Female		5	71.4%
Average age	56.7 years		(21–80)	34 years			(23–45)
Disease	NHL	8	33.3%				
	Myeloma	6	25%				
	AML	4	16.7%				
	ALL	2	8.3%				
	HL	1	4.2%				
	CML	1	4.2%				
	Other (JMML, ET + SSc)	2	8.3%				
Vaccine received	Astra-Zeneca	8	33.3%	Astra-Zeneca		1	14.3%
	Pfizer	16	66.7%	Pfizer		6	85.7%
				**Time from last treatment (months)**			
Treatment	alloHSCT	8	33.3%	31.2	(5–96)		
	ASCT	3	12.5%	71	(2–204)		
	B-cell depleting +/− chemotherapy	8	33.3%	0	(all concurrent)		
	Plasma cell targeted therapy	2	8.3%	0	(all concurrent)		
	IMiDs	3	12.5%	3.2	(0–8)		

## Data Availability

The original contributions presented in this study are included in the article/Supplementary Materials. Raw data supporting the conclusions of this article can be made available upon request to the corresponding author.

## Supplementary Materials

The following supporting information can be downloaded at: https://www.mdpi.com/article/10.3390/vaccines14030201/s1; Table S1: List of flow cytometry antibodies; Figure S1: Whole blood cytokine responses after SARS-CoV-2 vaccination; Figure S2: Serum cytokine responses after SARS-CoV-2 vaccination; Figure S3: Gating strategy for immunophenotyping of immune cell subsets in HM PBMC pre-vaccination; Figure S4: Immunophenotyping of immune cell subsets in HM pre-vaccination PBMC.

## Abbreviations

The following abbreviations are used in this manuscript:

HM	Haematological malignancies
AlloHSCT	Allogeneic haematopoietic stem cell transplantation
Tfh	T follicular helper cells
VST	Virus-specific T-cells
Ig	Immunoglobulin
S protein	Spike protein
PBMCs	Peripheral blood mononuclear cells
PHA	Phytohaemagglutinin
CBA	Cytometric bead array
PEA	Proximity extension assay
NPX	Normalised protein expression
CVM	Patients with haematological malignancies
NHL	Non-Hodgkin lymphoma
AML	Acute myeloid leukaemia
ALL	Acute lymphoblastic leukaemia
HL	Hodgkin lymphoma
CML	Chronic myeloid leukaemia
JMML	Juvenile myelomonocytic leukaemia
ET	Essential thrombocythemia
SS	Systemic sclerosis
ASCT	Autologous stem cell transplantation
IMiDs	Immunomodulatory drugs
CVH	Healthy controls
CVM-R	Immunosuppressed patients vaccine responders
CVM-NR	Immunosuppressed patients vaccine non-responders
IL	Interleukin
IFNγ	Interferon gamma
CXCL13	Chemokine (C-X-C motif) ligand 13
CRTAM	Class-I MHC-restricted T-cell-associated molecule
GZMA	Granzyme A
CCL19	C-C motif chemokine ligand 19
ANGPT2	Angiopoietin-2
ADA	Adenosine deaminase
Tph	T peripheral helper cell
CM	Central memory cell
EM	Effector memory cell
NKT	Natural killer T-cell
Treg	Regulatory T-cell
TNF	Tumour necrosis factor
WBA	Whole Blood Assay
CTL	Cytotoxic T Lymphocyte
EBV	Epstein–Barr Virus
pDCs	Plasmacytoid Dendritic cells
DCs	Dendritic cells
