# Supplementary material for: Prospective Biomarkers of SARS-CoV-2 Vaccine Seroconversion in Patients with Haematological Malignancies
Source: Vaccines (Basel). 2026 Feb 25;14(3):201. doi: 10.3390/vaccines14030201 (PMC13030433; doi:10.3390/vaccines14030201)
Supplement: Supplementary file 1 [file vaccines-14-00201-s001.zip › vaccines-4056185-supplementary.pdf]

Table S1. List of flow cytometry antibodies.

Antibody	Fluorochrome	Clone	Catalogue No.	Supplier
**CD45RA**	BUV395	5H9	740315	BD Biosciences
**CD16**	BUV496	3G8	612944	BD Biosciences
**CCR5**	BUV563	2D7	741401	BD Biosciences
**CD11c**	BUV661	B-ly6	612967	BD Biosciences
**CD56**	BUV737	NCAM16.2	612766	BD Biosciences
**CD8**	BUV805	SK1	612889	BD Biosciences
**CCR7**	BV421	G043H7	353208	Biolegend
**CD123**	Super Bright 436	6H6	62-1239-42	eBioscience
**CD161**	eFluor 450	HP-3G10	48-1619-42	eBioscience
**IgD**	BV480	IA6-2	566138	BD Biosciences
**CD3**	BV510	OKT3	317332	Biolegend
**CD20**	Pacific Orange	HI47	MHCD2030	Invitrogen
**IgM**	BV570	MHM-88	314517	Biolegend
**IgG**	BV605	G18-145	563246	BD Biosciences
**CD28**	BV650	CD28.2	302946	Biolegend
**CCR6**	BV711	G034E3	353436	Biolegend
**CXCR5**	BV750	RF8B2	747111	BD Biosciences
**PD-1**	BV785	EH12.2H7	329929	Biolegend
**CD141**	cFluor B515	M80	R7-20113	Cytek
**CD57**	cFluor B532	HNK-1	R7-20656	Cytek
**CD14**	cFluor B548	63D3	R7-20115	Cytek
**CD45**	PerCP	H130	MHCD4531	Invitrogen
**CD11b**	PerCP-Cy5.5	ICRF44	301328	Biolegend
**TCRgd**	PerCP-eFluor 710	B1.1	46-9959-42	Invitrogen
**CD25**	PE	BC96	12-0259-42	eBioscience
**CD4**	cFluor YG584	SK3	R7-20041-100T	Cytek
**CD24**	PE-eFluor 610	SN3	61-0247-41	eBioscience
**CD95**	PE-Cy5	DX2	15-0959-42	eBioscience
**CXCR3**	PE-Cy7	CEW33D	25-1839-42	eBioscience
**CD27**	APC	O323	17-0279-42	eBioscience
**CD1c**	Alexa Fluor 647	L161	331510	Biolegend
**CD19**	Spark NIR 685	HIB19	302270	Biolegend
**CD127**	cFluor R720	A019D5	RC-00009	Cytek
**HLA-DR**	APC-eFluor 780	L243	47-9952-42	eBioscience
**CD38**	APC-Fire 810	HIT2	303550	Biolegend

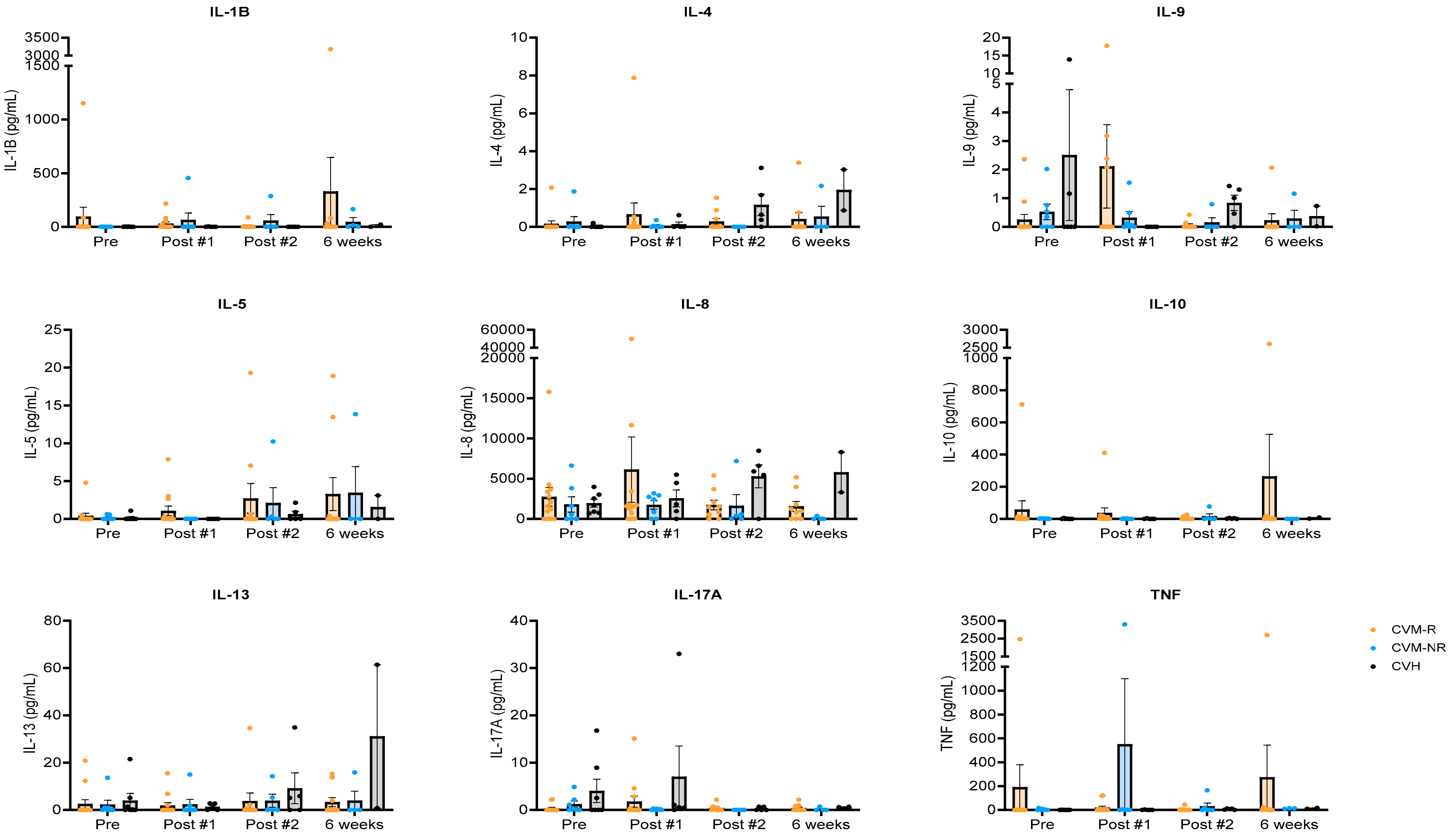

Figure S1. Whole blood assay cytokine analysis.

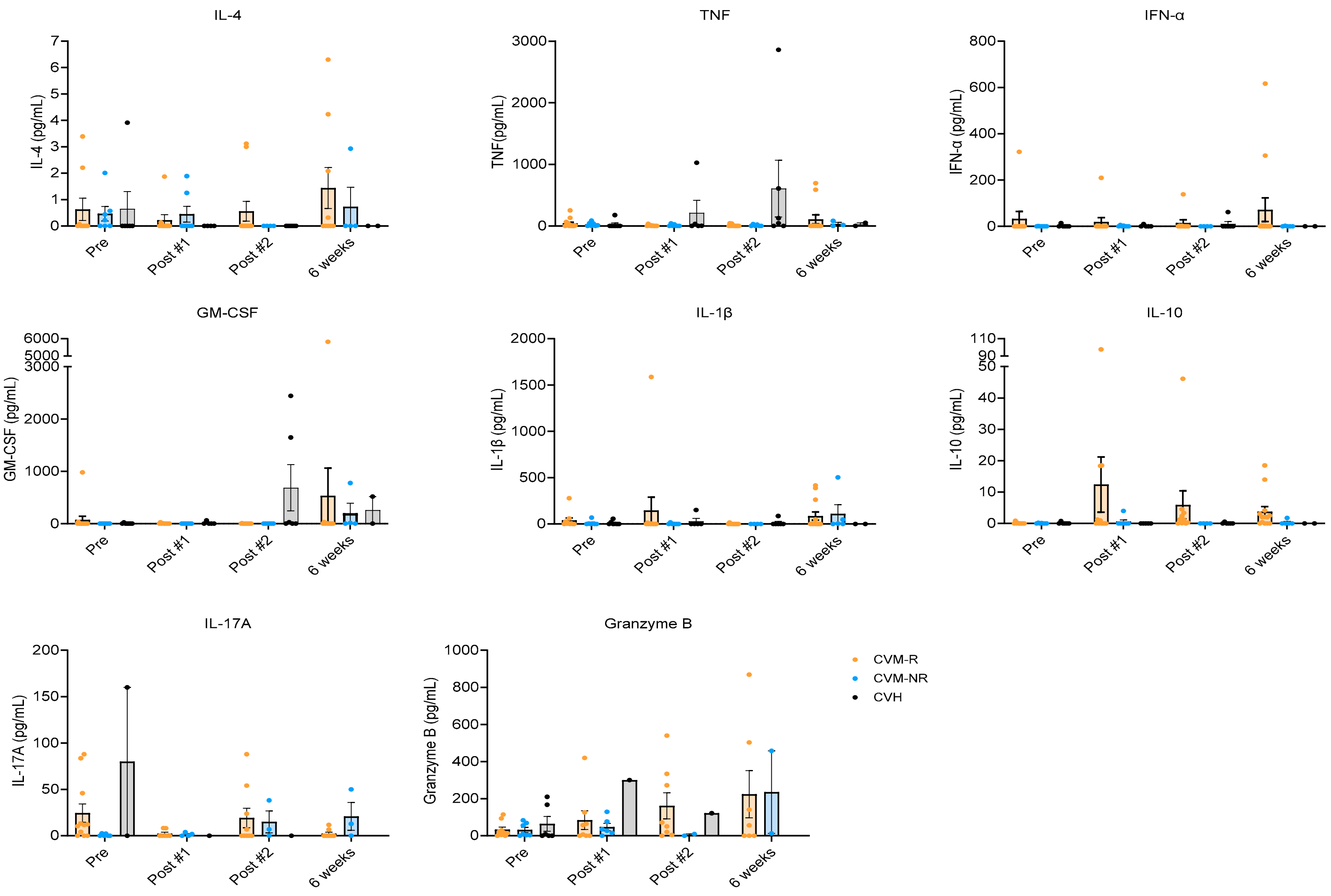

Figure S2. Serum cytokine responses after SARS-CoV-2 vaccination. IL-4, TNF, IFNα, GM-CSF, IL-1β, IL-10, IL-17A and Granzyme B responses in in serum of CVM patients. Statistical significance was determined by a multiple unpaired t test. Data presented as mean ± SEM.

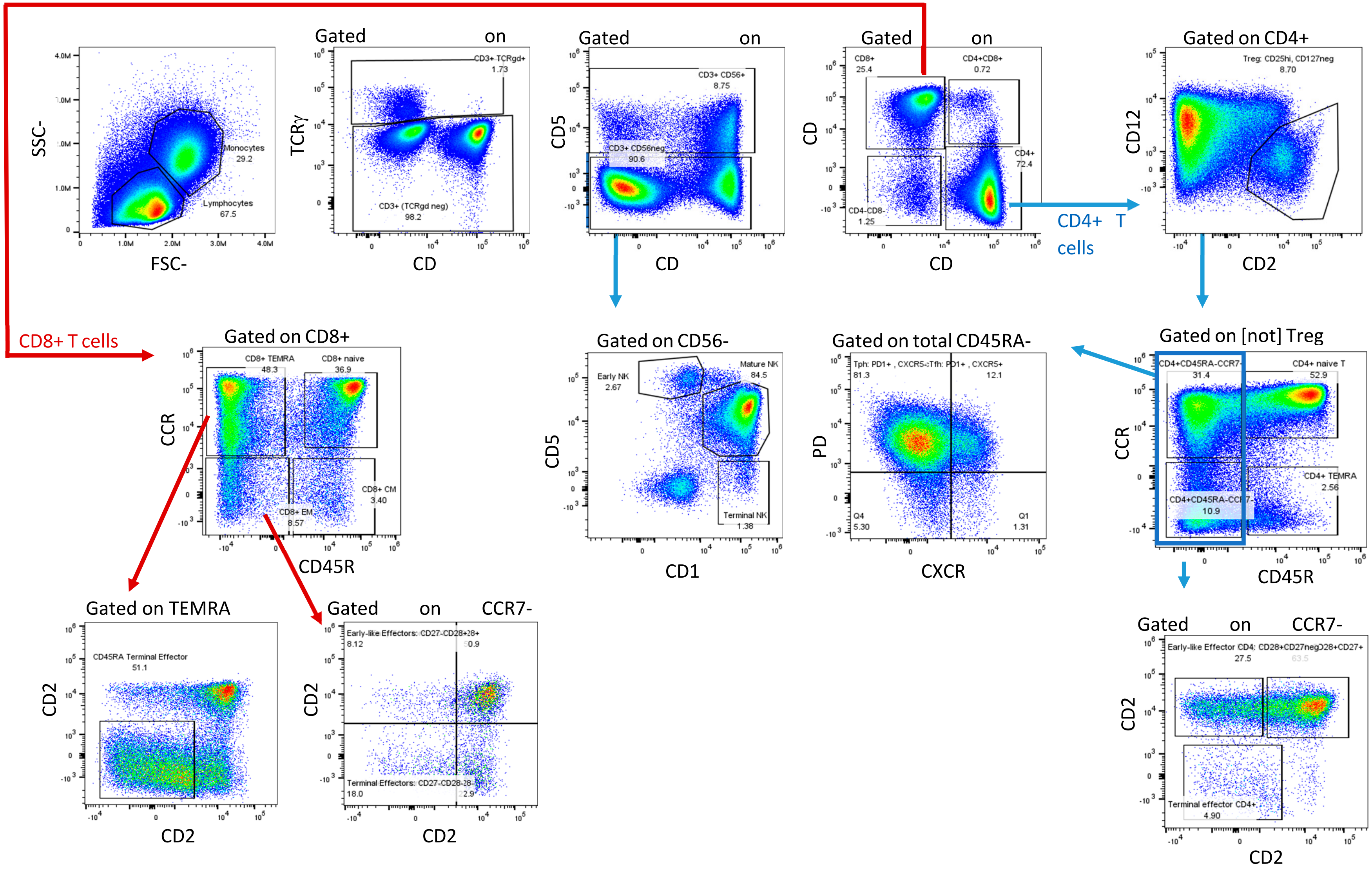

Figure S3. Gating strategy for Immunophenotyping of immune cell subsets in HM PBMC pre-vaccination.

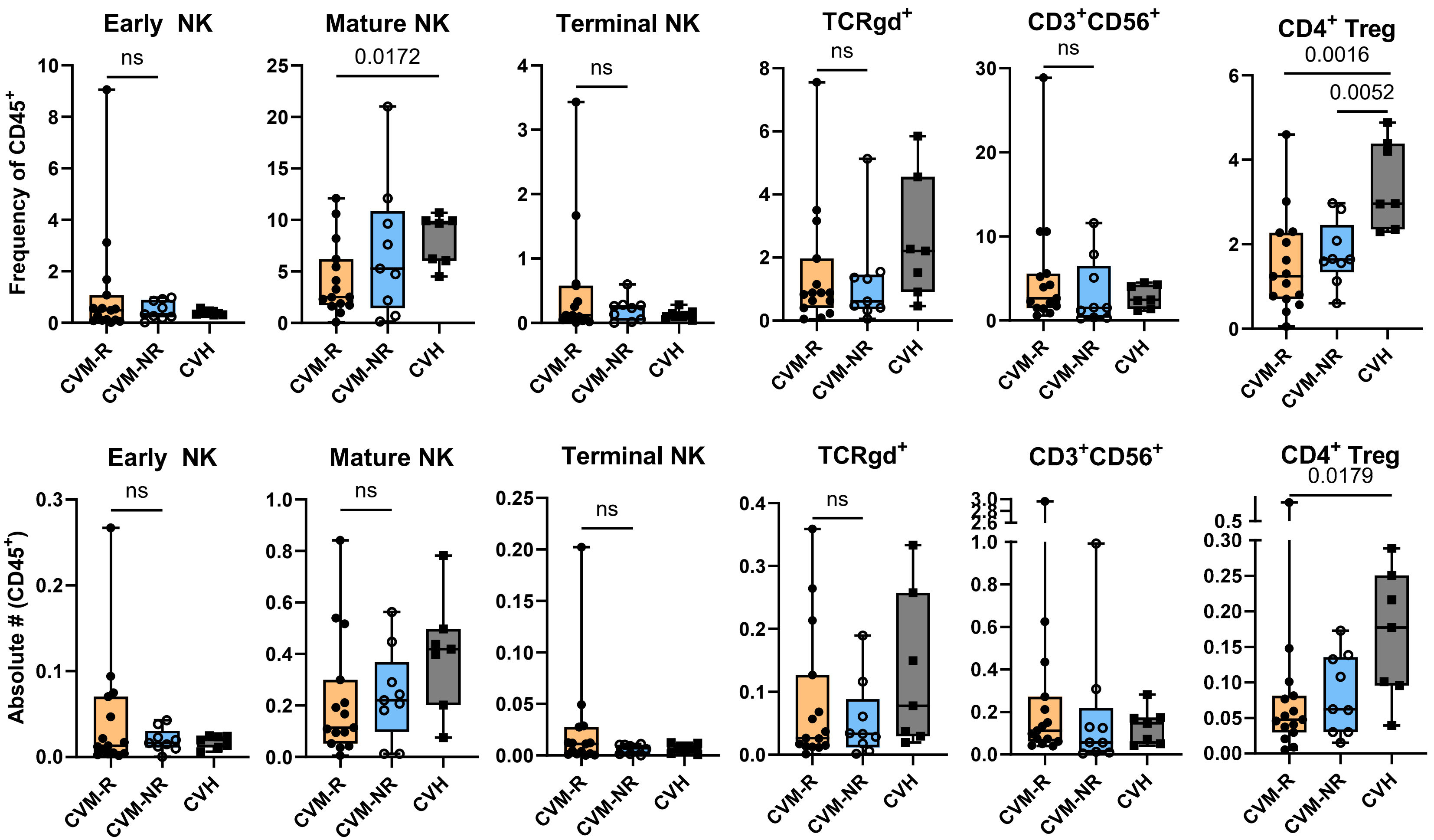

Figure S4. Immunophenotyping of immune cell subsets in HM pre-vaccination PBMC. Box and whisker plots displaying frequency (%) and absolute number (#) of Natural Killer (NK) subsets including: Early NK (CD56brightCD16-); Mature NK (CD56dimCD16+); Terminal NK (CD56-

CD16+) and T cell subsets including: TCRgd (CD3+TCRgd+); NKT (CD3+CD56+); Treg (CD4+CD25+CD127-). Statistical significance was determined by Mann-Whitney U Test.
